# microRNA dysregulation in polyglutamine toxicity of TATA-box binding protein is mediated through STAT1 in mouse neuronal cells

**DOI:** 10.1186/s12974-017-0925-3

**Published:** 2017-08-03

**Authors:** Reema Roshan, Ashwani Choudhary, Aksheev Bhambri, Bhawani Bakshi, Tanay Ghosh, Beena Pillai

**Affiliations:** 1 CSIR-Institute of Genomics and Integrative Biology, Mathura Road, New Delhi, 110025 India; 2 Academy of Scientific and Innovative Research (AcSIR), Mathura Road, Delhi, 110025 India; 3 Present address: Indian Council of Medical Research, New Delhi, India; 4 Indian Institute of Science, Centre for Neuroscience, Bangalore, 560012 Karnataka India; 5 Wellcome-Medical Research Council Cambridge Stem Cell Institute, Department of Clinical Neurosciences,, University of Cambridge, Cambridge, UK

**Keywords:** Polyglutamine diseases, SCA17, IFN-ϒ, STAT1

## Abstract

**Background:**

Polyglutamine diseases constitute a class of neurodegenerative disorders associated with expansion of the cytosine-adenine-guanine (CAG) triplet, in protein coding genes. Expansion of a polyglutamine tract in the N-terminal of TBP is the causal mutation in SCA17. Brain sections of patients with spinocerebellar ataxia 17 (SCA17), a type of neurodegenerative disease, have been reported to contain protein aggregates of TATA-binding protein (TBP). It is also implicated in other neurodegenerative diseases like Huntington’s disease, since the protein aggregates formed in such diseases also contain TBP. Dysregulation of miR-29a/b is another common feature of neurodegenerative diseases including Alzheimer’s disease, Huntington’s disease, and SCA17. Using a cellular model of SCA17, we identified key connections in the molecular pathway from protein aggregation to miRNA dysregulation.

**Methods:**

Gene expression profiling was performed subsequent to the expression of TBP containing polyglutamine in a cellular model of SCA17. We studied the expression of STAT1 and other interferon-gamma dependent genes in neuronal apoptosis. The molecular pathway leading to the dysregulation of miRNA in response of protein aggregation and interferon release was investigated using RNAi-mediated knockdown of STAT1.

**Results:**

We show that the accumulation of polyglutamine-TBP in the cells results in interferon-gamma release which in turn signals through STAT1 leading to downregulation of miR-29a/b. We propose that the release of interferons by cells harboring toxic protein aggregates may trigger a bystander effect resulting in loss of neurons. Interferon-gamma also led to upregulation of miR-322 although this effect is not mediated through STAT1.

**Conclusions:**

Our investigation shows that neuroinflammation could be an important player in mediating the transcriptional dysregulation of miRNA and the subsequent apoptotic effect of toxic polyglutamine-TBP. The involvement of immunomodulators in polyglutamine diseases holds special therapeutic relevance in the light of recent findings that interferon-gamma can modulate behavior.

## Background

Polyglutamine diseases are caused by expansion of polymorphic cytosine-adenine-guanine (CAG) triplets in protein coding regions of the genome resulting in aggregation-prone proteins. Accumulation of nuclear protein aggregates has been demonstrated in several polyglutamine diseases [1–4]. These diseases show some common features like increased severity in patients with longer expansions and anticipation, i.e., an earlier age of onset in subsequent generations [4]. However, each of the known polyglutamine diseases results in neuronal cell death in distinct parts of the brain. The pathological range of the causative triplet repeats expansions differ widely and occur in genes with no apparent functional overlap. For instance, Huntington’s disease is caused by expansion of the polyglutamine stretch beyond 35 repeats, in the protein Huntingtin, resulting in striatal and cortical neuronal cell death [5], while Spinocerebellar Ataxia 17 is caused by mutations of a stretch of more than 42 polyglutamines in the general transcription factor, TATA-binding protein, resulting in loss of cerebellar neurons [6].

TBP, a general transcription factor, has been found in the nuclear aggregates in postmortem brain tissue of patients of Huntington’s disease (HD) [7] and Spinocerebellar ataxias like (SCA1, SCA2, and SCA3) [8]. TBP has also been found in aggregates of Alzheimer’s disease (AD) [9] as well as neuronal intranuclear hyaline inclusion disease (NIH-ID) [10], suggesting that besides its direct involvement in SCA17, TBP is also affected in other neurodegenerative diseases. Gene expression studies from other groups and ours have pointed to transcriptional dysregulation in polyglutamine diseases [11, 12].

We have previously shown that expression of polyglutamine-expanded TBP in mouse neuronal cells is associated with formation of intra-nuclear protein aggregates and cell death [12]. Expression profiling of microRNAs during this process revealed temporal changes that match in vivo findings in mice and patients [13]. The downregulation of miR-29a/b was a key step in the molecular pathway leading to apoptotic cell death since transient knockdown of the miRNA-induced partial cell death in cultured cells [13]. Brain specific, transient knockdown of the miRNA was sufficient to induce ataxia-like behavior in mice [14], while stable knockout of the miRNA resulted in smaller Purkinje cells with reduced dendritic arborization, locomotor impairment, and ataxia–distinctive features seen in SCA17 transgenic mice models [15]. The downregulation of miR-29a/b is not restricted to SCA17 but is a general feature of several neurodegenerative diseases including Alzheimer’s disease, Huntington’s disease, and schizophrenia [16]. Besides neurodegenerative diseases, neuronal apoptosis is accompanied by downregulation of miR-29a/b during embryonic stages of brain development [17].

miR-29a/b plays a critical role in neuronal survival, but the events that modify the expression of these regulatory RNAs during neurodegeneration are not known. Identification of regulatory molecules that control the expression of miR-29a/b in neurons could provide insight into pathological mechanisms. Using the cellular model of SCA17, we explored the molecular pathway that leads to downregulation of miR-29a/b following the expression of polyglutamine containing TBP. We carried out gene expression profiling in this cellular model of SCA17 and found that a set of five co-regulated genes was induced in polyglutamine-TBP containing cells. The genes include STAT1, a key transcriptional regulator, and four genes that are known to be induced by interferon-gamma (IFN-γ). Here, we show that the induction of STAT1 is necessary and sufficient to cause the previously reported downregulation of miR-29a/b during neuronal apoptosis. Besides miR-29a/b, we show that the IFN-γ mediated signaling also resulted in upregulation of miRNA, miR-322 through a STAT1 independent mechanism. This study establishes a sequence of events from polyglutamine aggregation to transcriptional dysregulation through cell signaling and consequent loss of anti-apoptotic miRNA expression leading to cell death in cells expressing toxic polyglutamine-TBP.

## Methods

### Cell culture and transfection

Neuro-2a cells (mouse neuroblastoma cell line; ATCC number CCL-131; N2a) were maintained in Dulbecco’s modified Eagle’s medium (DMEM) (Invitrogen) supplemented with 10% fetal calf serum (Invitrogen), 2 mM L-glutamine (Sigma), 1 mM sodium pyruvate (Sigma), and antibiotic-antimycotic solution (100X stock) (Invitrogen) at 37 °C humidified incubator with 5% CO_2_. Transfection was performed using Amaxa nucleofection technology and following the manufacturer’s protocol. 10^6^ cells and 3 μg of plasmid DNA were used for transfection. Transfection efficiency was calculated by counting GFP positive cells, which came out to be ~50%.

### RNA isolation and microarray

Total RNA from Neuro-2a cells (24, 36, and 60 h post-transfection) was isolated using Trizol reagent following the manufacturer’s protocol. Microarray experiments were done using mouse Genome 430 2.0 (GeneChip). Briefly, 100 ng of total RNA was used for labeling reaction. Labeling, hybridization, and washing steps were performed following the manufacturer’s protocol.

### Data analysis

Microarray hybridization was carried out with three biological replicates for both 16Q-TBP and 59Q-TBP expressing cells. Background correction was performed and features flagged for low expression were removed before data was normalized using quantile normalization. For each probe, at every time point, a *t* test was used to test the null hypothesis that the expression level between 16Q-TBP and 59Q-TBP was unchanged. At the same time, the average intensity level for all replicate spots for every probe was calculated for 16Q-TBP and 59Q-TBP and the fold change was estimated. Probes for which the difference between samples was greater than 1.5 fold and at least one time point had a *t* test significance level less than 0.05/315 = 1.59 × 10^−4^ were selected.

### Transfection and luciferase assay

Neuro-2a cells, seeded in a 96-well plate at about 20,000 cells per well and grown for 24 h, were co-transfected with constructs as indicated in the results using Lipofectamine (Invitrogen) as per manufacturer’s protocol. Twenty-four hours after transfection, cells were lysed in 20 μl of passive lysis buffer (Promega). The luciferase assay was performed using a Dual-Luciferase assay kit (Promega), and luminescence was measured in a microplate Luminescence counter (Top count NXT PerkinElmer). For data analysis, Renilla luciferase was normalized to firefly luciferase.

### Cytochrome c assay

Cells were scraped, washed in 1XPBS, resuspended in 1× extraction buffer A containing 10 mM HEPES, pH 7.5, 200 mM mannitol, 70 mM sucrose and 1 mM EGTA (Sigma) and 2 mg per ml bovine serum albumin (Sigma) and kept on ice for 1 h. Cells were lysed by a B-type Dounce homogenizer, and homogenates were centrifuged at 4 °C in the subsequent steps to remove nuclei, debris, and mitochondria. To obtain the cytosolic fraction, supernatant was centrifuged at 15,000 g at 4 °C. The cytochrome c concentration in the cytosolic fraction was quantified by solid phase ELISA kit (Quantikine Rat/Mouse, R & D systems) according to the manufacturer’s protocol.

### Real-time PCR for miRNA-29a/b

For-real time PCR experiments, total RNA was isolated from Neuro-2a/HeLa cells using TRIzol reagent (Invitrogen) following the manufacturer’s protocol. Mouse brain was micro dissected into three different parts, cerebellum, cortex, and hippocampus. For real-time PCR, total RNA was isolated by crushing brain parts in TRIzol reagent using liquid nitrogen followed by manufacturer’s protocol. For cDNA synthesis and real-time PCR TaqMan assay (Applied Biosystems; Cat. No. 000412[miR-29a], 000413[miR-29b], 000430[miR-92]) specific for mature mmu-miR-29a, mmu-miR-29b and mmu-miR-92 was used. mmu-miR-92 was used as endogenous control. Relative quantification method was used for data analysis.

For Stat1, Usp18, Gbp3, Cxcl10, and Isg15, cDNA was prepared using gene-specific reverse primers and M-MuLV reverse transcriptase (NEB) at 42 °C for 1 h. Real-time PCR was performed using gene specific primers and SYBR green master mix (Roche). The sequences are given below:

**Table:** 

Gene	Forward primer	Reverse primer	Product size (bp)
Stat1	GCTGTGCCTCTGGAATGATG	CGGGAGCTCTCACTGAATCT	110
Usp18	CCTTGTCTGCTGCATTTCAA	TTCCGTGTGTGAGCTTTCAG	120
Gbp3	GCATCTCCTGTGGAGCTTTC	TCACTCCCTTCCTCAGCACT	105
Isg15	AAGAAGCAGATTGCCCAGAA	TCGCTGCAGTTCTGTACCAC	149
Cxcl10	TCCTTGTCCTCCCTAGCTCA	ATAACCCCTTGGGAAGATGG	124
18srRNA	CCTCCAATGGATCCTCGTTA	CTTTCGAGGCCCTGTAATTG	63

### Induction of IFN-γ

One hunderd units/ml of mouse recombinant IFN-γ was exogenously added to Neuro-2a cells. Briefly, 10^6^ cells were seeded 1 day prior to induction. Next day, 100 units of IFN-γ dissolved in PBS + 0.1%BSA was added per ml of media and kept at 37 °C. PBS + 0.1%BSA was used as control.

### Reporter assay for IFN-γ

Reporter assay was performed using Cignal GAS Reporter (luc) Kit: CCS-009 L (Qiagen) as per manufacturer’s protocol. Briefly, 20,000 cells were seeded in each well of a 96-well plate. Next day cells were transfected with 16Q-TBP/59Q-TBP constructs along with the GAS-responsive firefly luciferase construct along with Renilla luciferase expressing construct and kept at 37 °C for 24, 36, and 60 h. Post-transfection, dual-luciferase assay was performed using dual-luciferase kit (Promega).

### Spent media and neutralizing IFN-γ antibody experiments

Neuro-2a cells were transfected with 16Q-TBP and 59Q-TBP constructs and kept at 37 °C for 36 h. Post-transfection media from these cells was taken and put on untransfected Neuro-2a cells, these cells were further incubated at 37 °C for 24 h. For neutralizing IFN-γ experiments, similar transfections were done, and 36-h post transfection 2 μg/ml of IFN-γ antibody (abcam-ab10745) was added and kept at 37 °C for 12 h. Media from these cells was applied to untransfected Neuro-2a cells and kept for 24 h.

### Over-expression and knockdown of Stat1, miR-322

For over-expression of mouse Stat1, 2.5 kb of mouse Stat1 CDS was cloned under CMV promoter using pEGFPN3 plasmid (Promega). The sequence for primers is given below:

Stat1 FP: ATATCTCGAGCGGAGACAGCCCAGTAAGTC

Stat1 RP: ATATGGATCCCAGCASTGCTCAGCAAATGT

For knockdown experiments, 100 nM of Stat1siRNA (sigenome)/negative control siRNA was transfected into Neuro-2a cells for 48 h. ON-TARGET plus non-targeting siRNA (Thermo scientific D-001810-01) was used as negative control. miR-322 was cloned from mouse genomic DNA using primers flanking the pre-miRNA sequences. The amplified product of approximately 110 bp was cloned in pSilencer 4.1 CMV Neo Vector using BamHI and HindIII sites.

## Results

**Fig. 1:**
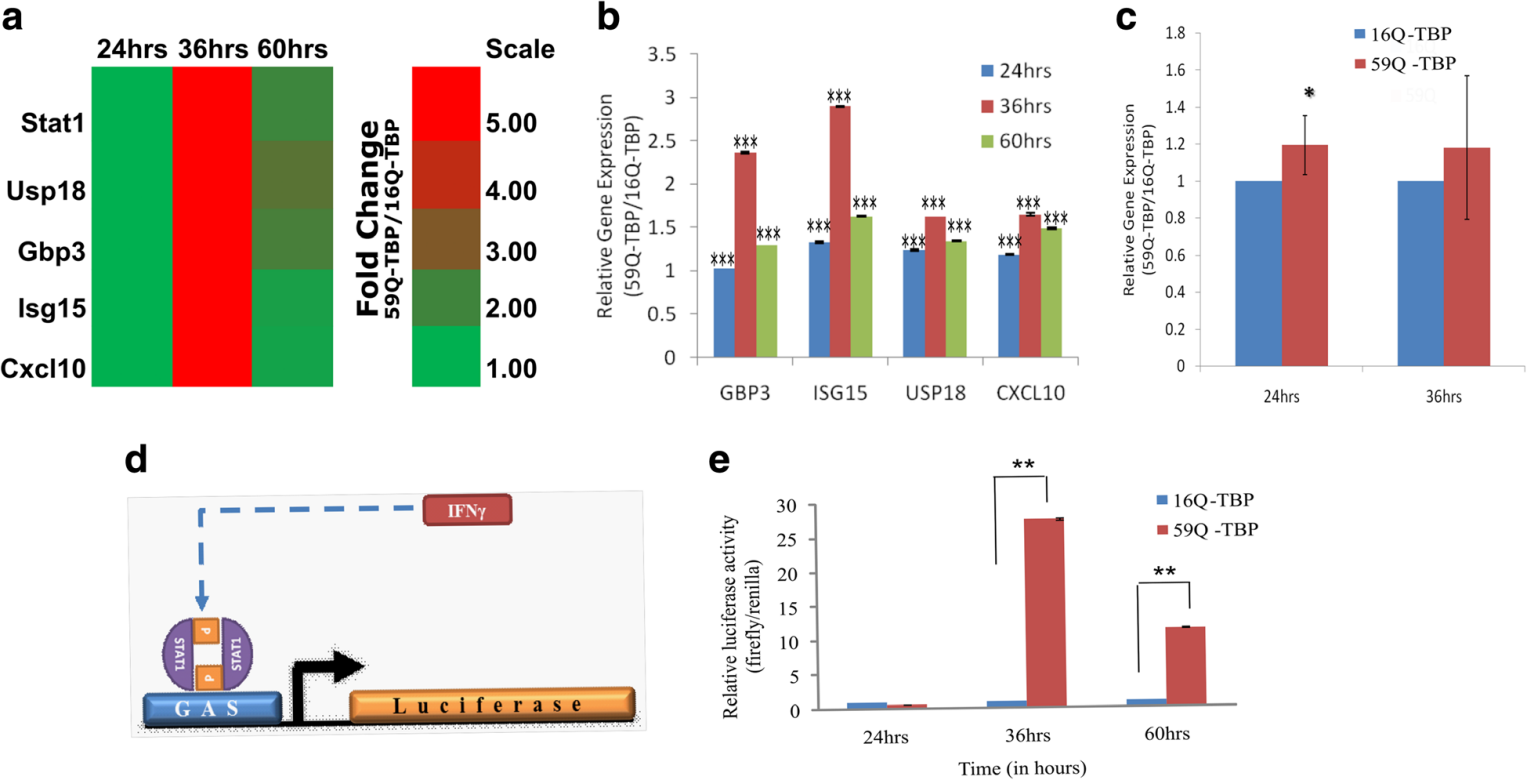
Differential expression of Stat1 and its dependent genes. Neuro-2a cells were transfected with 16Q-TBP or 59Q-TBP and total RNA compared by microarray analysis (**a**) or qRT-PCR (**b**, **c**) at indicated times (24, 36, and 60 h post transfection). Relative expression indicates fold change with respect to expression at 24 h post transfection. Reporter assay for IFN-γ dependent upregulation was studied by co-transfecting GAS promoter-luciferase reporter fusion plasmid (**d**) in Neuro-2a cells. Induced GAS promoter activity in the presence of 59Q–TBP (**e**). (*n* = 3; *p value <0.05; **p value ≤ 0.01; ***p value ≤ 0.001)

To understand the global gene expression changes occurring in SCA17 condition, we performed microarray experiments using the Affymetrix platform in the previously reported cellular model of SCA17. Neuro-2a cells were transiently transfected with two alternative constructs, with contain TBP, with a stretch of 16 (normal, 16Q) or 59 (pathogenic, 59Q) contiguous glutamine residues as a C-terminal fusion. As reported in our previous papers, we found that the pathogenic protein formed intra-nuclear aggregates, the expression of miR-29a/b was downregulated in a time-dependent manner, and the 59Q-TBP containing cells showed increased apoptosis [12–14]. In agreement with our previous studies using cDNA microarrays [12], we did not find a general reduction in global gene expression changes in polyglutamine-TBP containing cells. Both elevation and reduction of expression of specific genes at all three time points (24, 36, and 60 h) post transfection of expanded polyglutamine-TBP construct was observed. Interestingly, Signal Transducer and Activator of Transcription1 (Stat1) and its four dependent genes, Ubiquitin Specific Proteases18 (Usp18), Guanylate Binding Protein3 (Gbp3), Interferon Stimulated Gene15 (Isg15), and C-X-C motif chemokine10 (Cxcl10) were found to be significantly up regulated in Neuro-2a cells expressing polyglutamine-TBP condition (Fig. 1a).

To validate the result obtained from microarray analysis, real-time PCR was performed for all the five differentially expressed genes. Neuro-2a cells were transfected with plasmids expressing 16Q-TBP and 59Q-TBP, and qRT-PCR was done for five genes (Stat1, Usp18, Gbp3, Isg15, and Cxcl10) using gene specific primers (Fig. 1b). Stat1 mRNA was mildly induced at 24 h, while downstream Stat1-dependent genes such as Usp18, Gbp3, Isg15, and Cxcl10 were all induced at 36 h (Fig. 1b, c).

Gamma activating sequences (GAS) elements are upstream to genes that respond to IFNγ signaling by binding to activated STAT1 dimers. We checked the time-dependent effect of 59Q-TBP on a reporter construct with tandem GAS elements fused to a luciferase reporter (Fig. 1d). Cells with 59Q-TBP showed a strong induction of the luciferase reporter indicating active IFN signaling (Fig. 1e).

We then looked at differentially expressed genes identified in the microarray analysis and functionally classified them to assess genes involved in specific biological processes. Upregulated genes showed significant enrichment for interferon and immunity-related processes (Table 1) while downregulated genes remained unclassified with no significant enrichment for any biological process. Notably, IFN-γ induced protease, cytokines, and ubiquitin-like proteins were found to be increased in 59Q-TBP.

**Table 1:** List of differentially expressed interferon-related genes and log fold changes at 36 h in 59Q-TBP condition compared to 16Q-TBP

Gene	Function	Log_2_ (Fold Change)
Gbp3	Interferon gamma induced Guanylate binding protein	2.37
Tyki	Thymidine kinase induced by interferon in antiviral response	2.16
Ifih1	Interferon-induced helicase	2.12
Rsad2	Interferon-induced antiviral protein	2.12
Samd9l	Sterile alpha motif domain-containing protein 9-like	1.99
Isg15	Interferon-stimulated protein similar to ubiquitin like molecules	1.97
Rsad2	Interferon-induced antiviral protein	1.84
Oas1c	Interferon induced, involved in response to viral infection	1.81
Oasl2	Interferon induced, involved in response to viral infection	1.78
Igtp	Interferon gamma inducible GTP binding protein	1.69
Psmb9	Interferon gamma induced protease	1.68
Cxcl10	Interferon gamma induced cytokine	1.65
Stat1	Interferon induced transcriptional factor	1.63
Apol9A	Interferon induced apolipoprotein	1.58
Usp18	Interferon induced ubiquitin specific peptidases	1.54
Rtp4	Interferon-induced receptor transporter protein	1.53

**Fig. 2:**
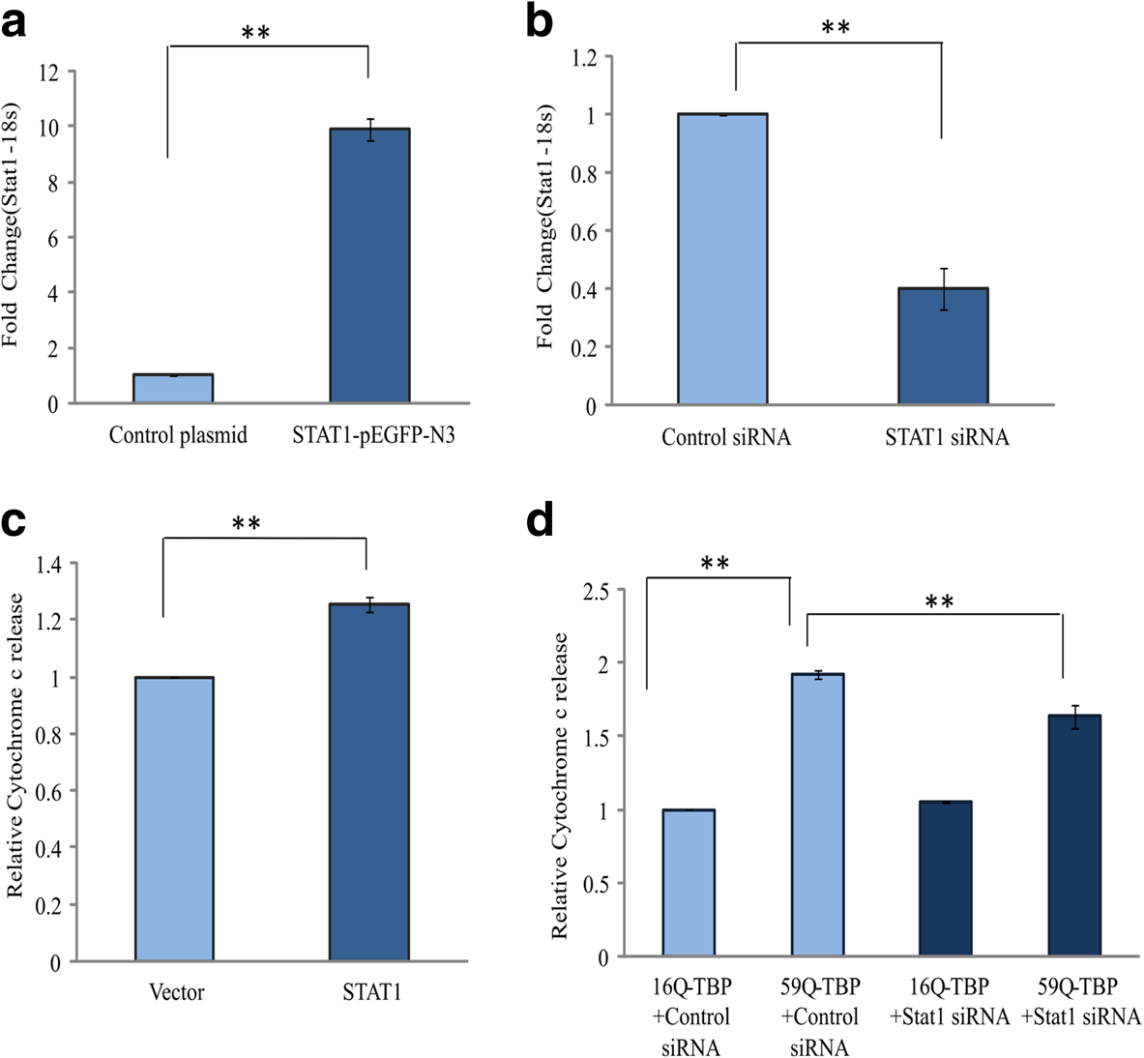
Stat1 over-expression leads to neuronal cell death. Neuro-2a cells were transfected with Stat1 expressing plasmid (STAT1-pEGFP-N3) or vector (control plasmid) (**a**) and negative control siRNA or siRNA against Stat1 (**b**). Real-time PCR was done to check the expression of Stat1 after 24 h under these conditions. Stat1 over-expression induced cytochrome c release while siRNA against Stat1 was sufficient to partially rescue the increased cytochrome c release (**c**, **d**). Neuro-2a cells were transfected with Stat1 over expressing and control plasmid, 24 h post transfection, cytosolic protein was isolated, and cytochrome c assay was done (**c**). Cells were co-transfected with 16Q-TBP/59Q-TBP constructs along with control siRNA and siRNA against Stat1 for 48 h, and after isolation of cytosolic protein fraction cytochrome c assay was done. (*n* = 3; **p value ≤ 0.01)

To understand the relevance of the STAT1-dependent genomic response, we studied the effects of Stat1 over-expression and knockdown on cell death by measuring cytochrome c release. For over expression studies, the coding region of mouse Stat1 was cloned under CMV promoter while siRNA against Stat1 was used for knockdown experiments. The ectopic expression of STAT1 resulted in a 10-fold upregulation of the steady state levels of the STAT1 mRNA (Fig. 2a). The knockdown of Stat1, by a STAT1 siRNA, was confirmed by real-time PCR (Fig. 2b). Under these conditions, we found a 21% increase in cytochrome c release (Fig. 2c). The STAT1 siRNA led to 60% downregulation of Stat1 mRNA, 40 h post-transfection. We used the STAT1 siRNA to reverse the elevated STAT1 levels in cells expressing the pathogenic 59Q-TBP. The STAT1 siRNA could only partially (28%) reverse the apoptosis, as indicated by cytochrome c release (Fig. 2d).

**Fig. 3:**
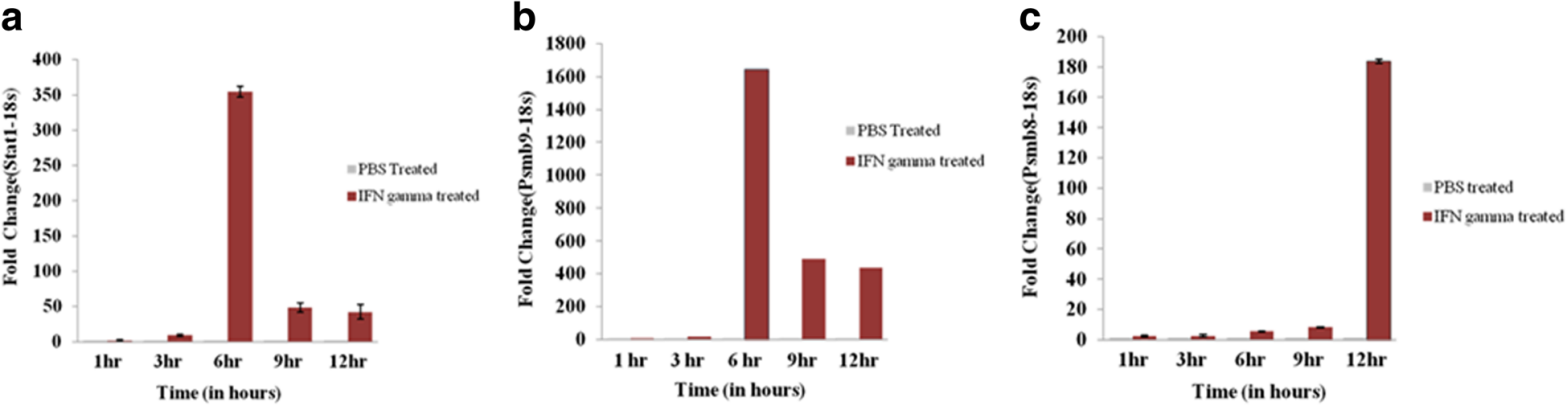
Neuro-2a responds to IFN-γ signaling. Neuro-2a treated with 100 units/ml of mouse recombinant IFN-γ or Phosphate buffered saline (PBS) were monitored using qRT-PCR at indicated times post-treatment to study the response of Stat1 (**a**), Psmb9 (**b**), Psmb8 (**c**) genes (*n* = 3)

We have previously reported the downregulation of miR-29a/b and miRNA profiling in cells expressing polyglutamine-TBP [13]. Besides the downregulation of miR-29a/b, we have also shown that miR-322 was upregulated concomitantly [13]. Under the same conditions, we also found that STAT1 and its dependent genes were upregulated. Therefore, we explored if the STAT1 over-expression was linked to the downregulation of miR-29a/b or upregulation of miR-322. We used IFN-γ to induce the expression of STAT1 in Neuro-2a cells, independent of polyglutamine-TBP. To establish the effect of IFN-γ induction in cells, the mRNA levels of genes such as Stat1 (Fig. 3a), Proteasome subunit beta type-9 (Psmb9) (Fig. 3b), and Proteasome subunit beta type-8 (Psmb8) (Fig. 3c) which are known markers of IFN-γ stimulation were measured. All the three genes were found to be significantly upregulated upon treatment of Neuro-2a cells with IFN-γ, compared to control treated cells confirming the successful induction as well as downstream signaling via this cytokine (Fig. 3) in Neuro-2a cells.

**Fig. 4:**
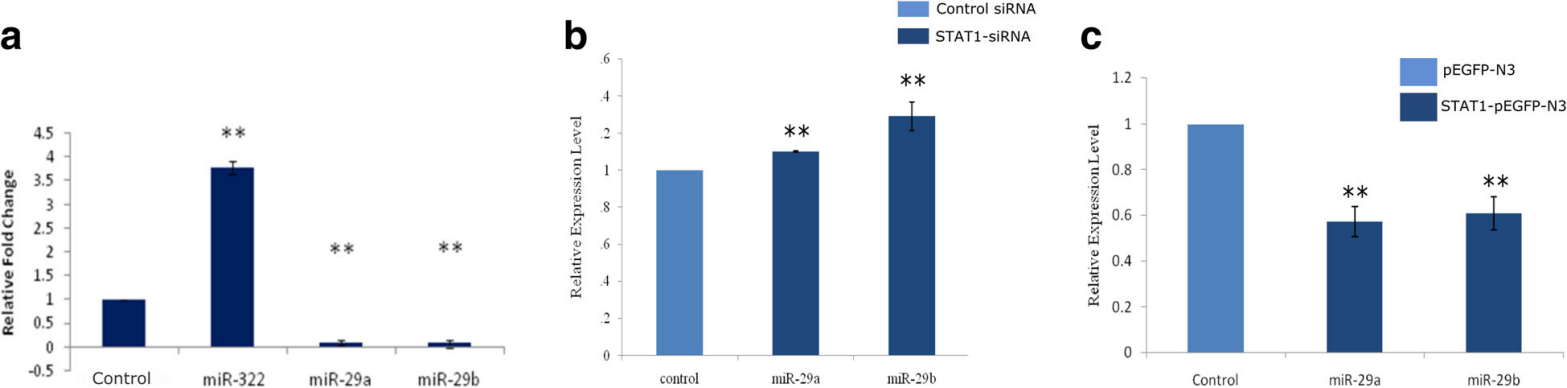
Neuro-2a cells modulate miRNA expression in response to IFN-γ treatment. **a** Neuro-2a cells treated with IFN-γ (100 units/ml) induced mmu-miR-322 and repressed mmu-miR-29a/b expression. **b** Stat1 knockdown using siRNA leads to upregulation of miR-29a/b. Neuro-2a cells were treated for 24 h with non-targeting siRNA or Stat1 siRNA. **c** Stat1 over-expression leads to decrease in miR-29a/b expression (*n* = 3; **p value ≤0.01)

After verifying that STAT1 and several interferon gamma dependent genes were induced as expected, we checked the effect of IFN-γ treatment on the microRNAs, mmu-miR-29a/b using qRT-PCR. We found that mmu-miR-29a/b was downregulated following IFN-γ treatment while mmu-miR-322 was upregulated (Fig. 4a). We knocked down Stat1 expression using siRNA against Stat1 mRNA and monitored the effect on miR-29a/b (Fig. 4b). As expected, over-expression of Stat1 under a strong CMV promoter resulted in the downregulation of both miR-29a and miR-29b (Fig. 4c), thus establishing that miR-29a/b is under direct transcriptional repression by STAT1. To confirm that STAT1 was required for the downregulation of miR-29a/b in polyglutamine-TBP cells, we simultaneously transfected the polyglutamine constructs and siRNA against STAT1. The siRNA against Stat1 resulted in 48% reduced expression of Stat1. Under these Stat1-depleted conditions, miR-29a/b was not significantly downregulated in 59Q-TBP cells, compared to 16Q-TBP (Average fold change of miR-29a = 1.07 ± 0.19; *p* value = 0.48; *n* = 3; miR-29b = 1.1 ± 0.32; *p* value = 0.38, *n* = 3).

**Fig. 5:**
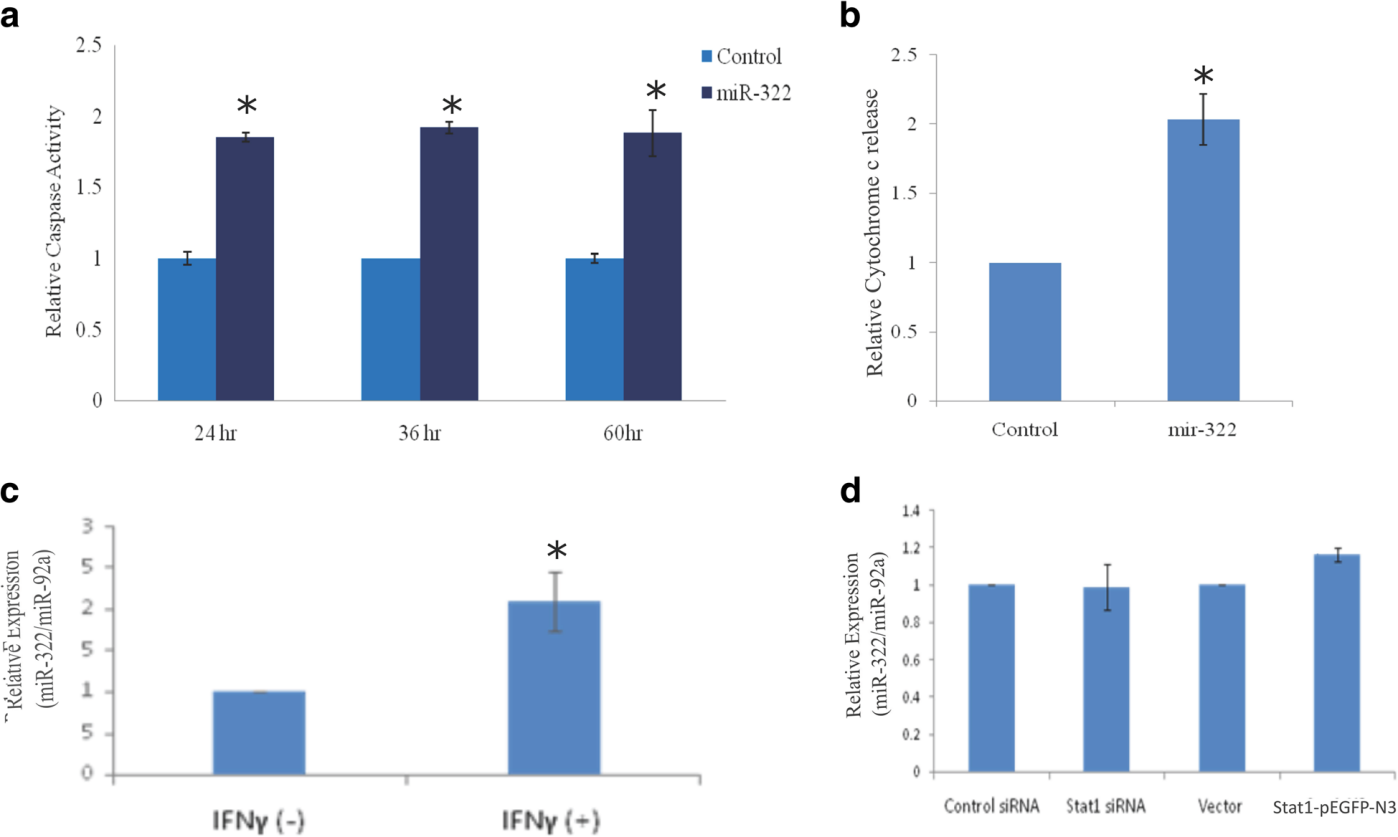
miR-322 over-expression can induce apoptosis in Neuro-2a cells: (**a**) The caspase-3 activity in Neuro-2a cells transfected with vector (control) or miR-322 clone (miR-322) was monitored at the time indicated (24, 36, 60 h post transfection). At 24 h, under the same conditions (**b**) relative cytochrome c release was elevated. IFN-γ treatment also led to the induction of miR-322 levels in qRT-PCR based assay (**c**). Stat1 knockdown (by Control or Stat1 siRNA) and over-expression (by Vector or Stat1-pEGFP-N3) has no significant effect on expression of miR-322 (**d**)

We have previously reported that miR-322 was induced in polyglutamine-TBP expressing cells [13]. To test if miR-322 affects apoptotic cell death triggered by polyglutamine toxicity, we cloned and over-expressed the pre-miR-322. We found that the over-expression of miR-322 resulted in elevated caspase 3 activity and cytochrome c release indicating increased apoptosis (Fig. 5a, b). Although polyglutamine-TBP and IFN-γ treatment independently led to the induction of miR-322 (Fig. 5c), over-expression and knockdown of STAT1 had no effect on miR-322 expression (Fig. 5d) in the cells, even when done in the background of TBP-59Q and TBP-16Q (Average fold change = 1.01 ± 0.18; *p* value = 0.9). We reasoned that IFN-mediated induction of miR-322 was not mediated through STAT1. This was also corroborated by the absence of GAS sites upstream to miR-322. Further, it agrees well with our finding that siRNA-mediated mitigation of STAT1 could only partially rescue the apoptotic effect of polyglutamine-TBP.

**Fig. 6:**
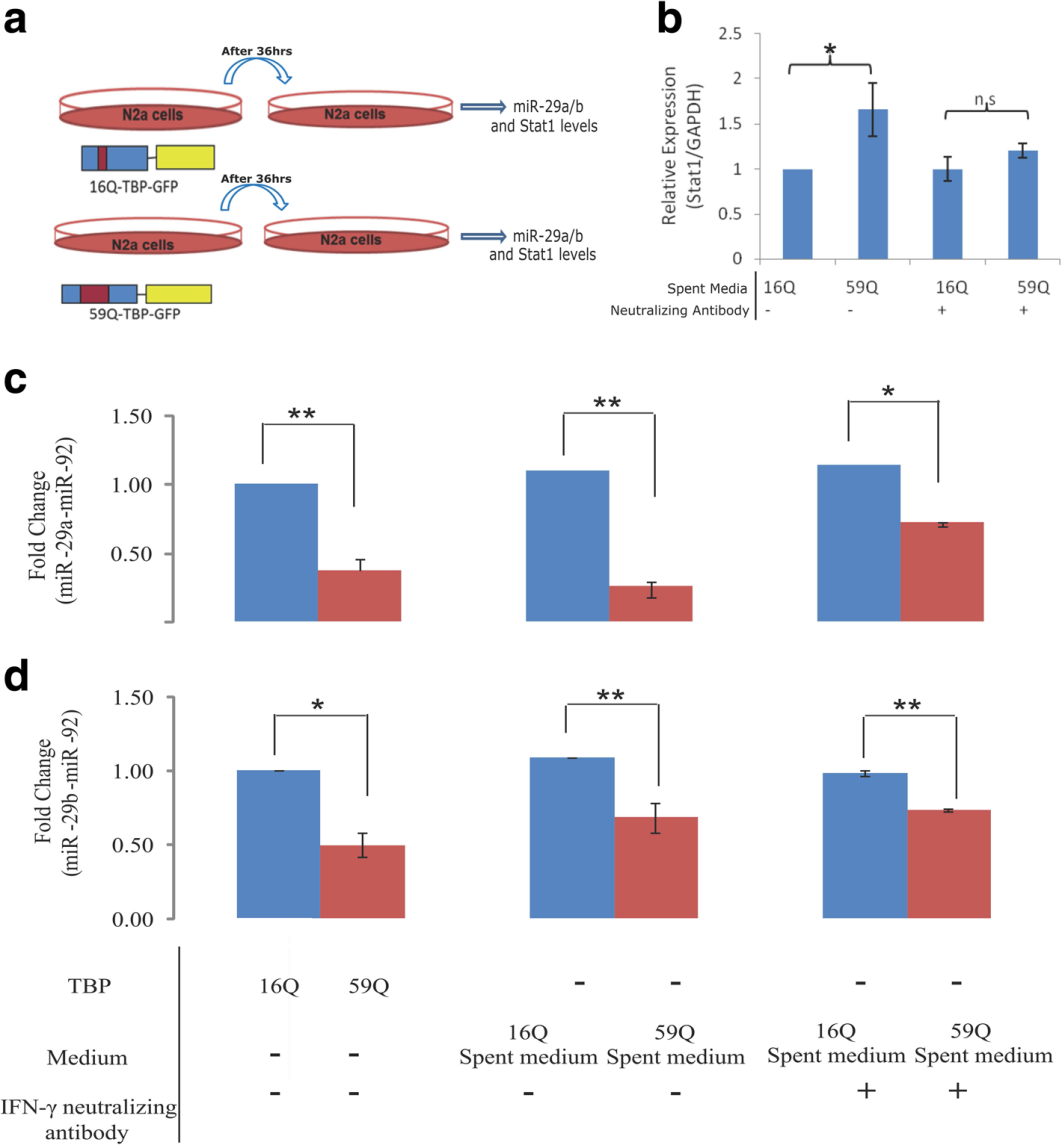
Spent media from 59Q-TBP cells carry factor(s) that modulate the levels of miR-29a/b. Neuro-2a cells were transfected with 16Q-TBP (16Q) or 59Q-TBP (59Q) and the supernatant media collected after 36 h (**a**). Neuro-2a cells were treated with this spent media (− in **b**, middle panel, **c-d**) or incubated with neutralizing antibody against IFN-γ before treatment on cells (+ in **b**, right panel, **c-d**). STAT1 (**b**) and miR-29a/b (**c-d**) levels were monitored using qRT-PCR and normalized to Gapdh (**b**) and miR-92a (**c-d**). (*n* = 3; * *p* value ≤0.01; ** *p* value ≤0.001)

Next we sought to identify events upstream to STAT1 induction in polyglutamine-TBP mediated miRNA dysregulation. We had noticed that the mRNA and miRNA dysregulation, be it induction of STAT1 or miR-322 and downregulation of miR-29a/b, were all reverted to normal levels following a change of media, suggesting that a secreted factor is responsible for the mRNA and miRNA expression changes. To test this hypothesis directly, we treated Neuro-2a cells with the spent media collected from 16Q or 59Q-TBP expressing cells (Fig. 6a). Media collected from 59Q-TBP-containing cells that were undergoing apoptosis could also induce STAT1 expression (Fig. 6b), downregulation of miR-29a/b expression in Neuro-2a cells (Fig. 6c, d). Since this effect was not seen in spent media of 16Q-TBP cells, we reasoned that polyglutamine expansion results in secretion of the factor into the surrounding media, which is in turn may be sufficient to trigger cell death, even in cells that have never expressed polyglutamine-TBP. Neutralizing antibodies against IFN-γ could suppress the effect of the spent media, partially restoring miR-29a but not miR-29b, suggesting that IFN-γ is the likely signaling factor released by polyglutamine-TBP containing neurons.

Taken together, our results suggest that neuronal cells expressing polyglutamine-TBP release interferons into the surrounding milieu which through a cascade of signaling events triggers the induction of STAT1 and subsequent downregulation of miR-29a/b. The interferon signaling also leads to the upregulation of miR-322, another dysregulated miRNA in polyglutamine-TBP cells, but this is independent of STAT1. Exogenously added interferon-gamma in the medium, spent media from the apoptotic cells or STAT1 over-expression, are sufficient to trigger miR-29a/b down regulation and apoptosis.

## Discussion

Here, by integrating the gene expression changes in mRNA and miRNA during polyglutamine-TBP mediated toxicity, we present a holistic view of molecular changes that potentially occur in SCA17. It is proposed that polyglutamine-TBP protein on aggregation induces interferon production in neurons. This interferon perhaps in conjugation with factors released by glia induces STAT1 and lead to downstream gene expression changes. A key effect of STAT1 induction is the repression of miR-29a/b that leads to previously established cascade of events marked by over-expression of apoptotic genes including Vdac1, Bace1, Puma, and Bak. IFN-γ neutralization in the spent media rescues miR-29a level but not miR-29b level. We concur that steady state levels of miR-29b seem to be recalcitrant to the spent media and the neurtralization of IFN therein. This is perhaps because miR-29 isoforms are coded for by two genes—the first encodes miR-29a and b-1 (Human: chr7:130,876,567-130,877,750; Mouse: chr6:31,062,609-31,063,227) and second encodes miR-29b and c (Human: chr1:207,801,476-207,802,623; Mouse: chr1:195,036,808-195,037,705). Thus, the miR-29b levels detected in the qRT-PCR are a combined effect of the two miR-29b genes. Moreover, miR-29b is known to be nuclear-localized and have distinct turnover dynamics that may render it recalcitrant to the effect of the neutralizing antibody. Although predicted STAT1 binding sites were noticed upstream to the miR-29a/b1 gene, we could not unequivocally demonstrate the direct binding of STAT1 at these sites. In publicly available ENCODE data, enrichment of STAT1 in the hsa-miR-29a/b promoter region is evident [18, 19].

STAT1-Dependent Genomic Response of Neurons has been studied extensively in the context of viral infections that lead to neurodegeneration [20]. STAT1 is also known to be involved in brain injury by regulating expression of proteins which contribute to cell death [21] (Takagi et al., [22]). However, the role of STAT1-mediated gene regulation has not been previously implied in inherited neurodegenerative diseases. Our studies show that, the downregulation of miR-29a/b, a common event in many neurodegenerative diseases like Alzheimer’s disease [23], Huntington’s disease [24], and Spino-cerebellar ataxia17 [16] is under the regulation of STAT1.

STAT1 is a key transcriptional mediator of interferon signaling pathways, leading to upregulation at the vast majority of target promoters, but can also switch to a repressive role at some promoters in association with specific partners that alter the specificity of heterodimeric STAT1 [25, 26]. In our study, the effect of interferon was only partially mediated through STAT1 suggesting that there are yet unknown players in this cellular pathway that connect polyglutamine toxicity to anti-apoptotic miRNAs.

Interferon-gamma is known to be intricately involved with miR-29a/b in a feedback regulatory loop in immune cells [27, 28]. However, the role of interferon signaling and its effects on miR-29a/b regulation in neuronal cells have not been studied before. Our results suggest that polyglutamine toxicity can trigger inter-cellular signaling by interferons resulting in two levels of response. Firstly, the secreted cytokines can bring about transcriptional changes in the cells carrying the polyglutamine protein. More interestingly, the cytokines can also lead to a bystander effect on other neurons. Speculating further, in the patient brain, neuron-glia crosstalk might lead to a further exacerbation of the bystander effect. A response originally evolved for destroying cells with a toxic build-up of protein aggregates perhaps triggers an uncontrolled bystander effect leading to the loss of a larger number of neurons.

IFN signaling in the brain was originally studied in the context of viral infections which trigger the release of interferons [29]. Once activated by interferons, STAT1 homodimer enters the nucleus and binds to cis-regulatory elements known as GAS elements containing a consensus sequence TTCN(2-4)GAA, to initiate or suppress transcription of IFN-regulated genes [30, 31]. In Alzheimer’s disease, the involvement of IFN-γ is well established [32], although ambiguous, since IFN-γ infusion shows both enhanced neurogenesis and aggravated pathology in transgenic mice models [33–35]. It is pertinent to note that downregulation of miR-29a/b, perhaps in response to IFN-γ, has been reported in Alzheimer’s disease pathology both in animal models and in patients [23, 36]. Notably, interferons are used as therapeutic agents in the treatment of auto-immune diseases of the central nervous system, for instance, multiple sclerosis [37, 38]. IFN-γ is one of the primary cytokines produced by CD8+T cells [39] as well as microglia in brain in response to stroke [40], brain injury [41], as well as in multiple sclerosis [42]. Recently, Filipino et al. have suggested that interferon signaling may be closely associated with neurological and psychiatric deficits seen in pathological conditions [43]. At the molecular level, they also showed conclusively that neurons and microglia express receptors to IFN-γ. The receptors allow the cells to respond to IFN-γ by increasing number of c-fos positive cells in mouse cortex [43]. Our results in the context of these in vivo findings highlight the need for studying IFN signaling in specific regions of well-established transgenic mice models of polyglutamine diseases.

## Conclusions

Gene expression profiling revealed STAT1 and four genes namely Usp18, Gbp3, Isg15, and Cxcl10 were upregulated in polyglutamine-TBP conditions. Integrating changes in mRNA and miRNA expression, we found that interferon signaling is critical to the downregulation of miR-29a/b and upregulation of miR-322. Our results strongly indicate that release of interferons by neuronal cells expressing polyglutamine-TBP leads to induction of STAT1 and consequent downregulation of miR-29a/b by a series of cell signaling events. On the other hand, interferon-mediated upregulation of miR-322 did not require STAT1, implying that further investigation will lead to identification of other key players in pathways connecting polyglutamine toxicity and the role of miRNAs in neuronal apoptosis. The involvement of interferon signaling in cellular mechanisms of polyglutamine-TBP mediated toxicity reveal the key role of neuro-inflammation in SCA17 pathogenesis.
